# Combined treatment of botulinumtoxin and robot-assisted rehabilitation therapy on poststroke, upper limb spasticity: A case report

**DOI:** 10.1097/MD.0000000000009468

**Published:** 2017-12-22

**Authors:** So Young Lee, Young Tae Jeon, Bo Ryun Kim, Eun Young Han

**Affiliations:** Department of Rehabilitation Medicine, Jeju National University Hospital, Jeju National University School of Medicine, Jeju, Republic of Korea.

**Keywords:** botulinumtoxin, dexterity, robot, spasticity, stroke

## Abstract

**Rationale:**

Spasticity is a major complication after stroke, and botulinumtoxin A (BoNT-A) injection is commonly used to manage focal spasticity. However, it is uncertain whether BoNT-A can improve voluntary motor control or activities of daily living function of paretic upper limbs. This study investigated whether BoNT-A injection combined with robot-assisted upper limb therapy improves voluntary motor control or functions of upper limbs after stroke.

**Patient concerns:**

Two subacute stroke patients were transferred to the Department of Rehabilitation.

**Diagnoses:**

Patients demonstrated spasticity in the upper extremity on the affected side.

**Interventions:**

BoNT-A was injected into the paretic muscles of the shoulder, arm, and forearm of the 2 patients at the subacute stage. Conventional rehabilitation therapy and robot-assisted upper limb training were performed during the rehabilitation period.

**Outcomes:**

Manual dexterity, grip strength, muscle tone, and activities of daily living function were improved after multidisciplinary rehabilitation treatment.

**Lessons:**

BoNT-A injection in combination with multidisciplinary rehabilitation treatment, including robot-assisted arm training, should be recommended for subacute spastic stroke patients to enhance appropriate motor recovery.

## 1 Introduction

Upper limb spasticity is a common complication following stroke, occurring in 20% to 40% of stroke survivors.^[1]^ As upper limb spasticity, joint contractures, and pain limit the voluntary motor control of the arm and hand, the functions of which are essential for the activity of daily living (ADL), ADL dependencies, including hygiene, dressing, and positioning, can be exacerbated.^[2]^

Injection of botulinumtoxin A (BoNT-A), which is commonly used in the management of focal spasticity in the chronic phase of stroke, reduces muscle tone and passive range of motion. However, it is unclear whether BoNT-A can improve voluntary motor control or ADL functions of upper limbs.^[3]^

Recently, task-specific high-intensity training with a multidisciplinary team approach has become an important concept in stroke rehabilitation therapy, and robot-assisted arm training (RAT) has been shown to allow well tolerated and intensive task-specific repetitive training of the paretic arm.^[4]^ However, multidisciplinary rehabilitation therapies using RAT in combination with BoNT-A injection have rarely been applied to subacute poststroke spasticity. Thus, we report on 2 cases showing the beneficial effects of RAT in combination with BoNT-A injection on upper limb spasticity in the subacute phase of stroke.

## 2 Case report

The study was approved by the Ethics Committee of Jeju National University Hospital. Written consent was obtained from both patients.

### 2.1 Patient 1

**Figure 1:**
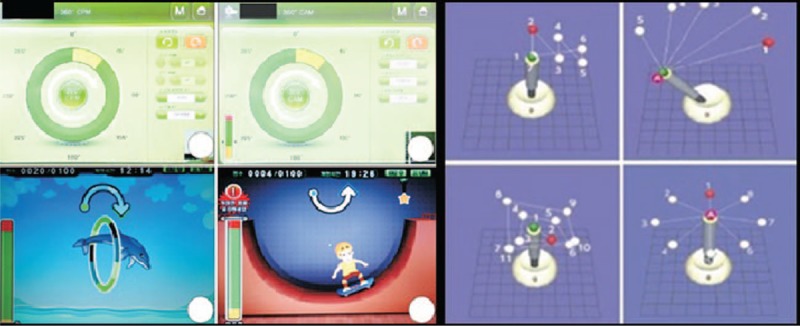
Programs of robotic-assisted arm training.

On April 27, 2016, a 37-year-old woman visited the emergency room of OO hospital because of hemiparesis in the right side. Brain magnetic resonance imaging revealed a left basal ganglia and corona radiata infarction. Antiplatelet medication (aspirin 300 mg/d, clopidogrel 75 mg/d), antihypertensive drugs (amlodipine 5 mg/d, fimasartan 60 mg/d, dichlozid 12.5 mg/d, nebivolol 5 mg/d), and atorvastatin (20 mg/d) were prescribed. On the 16th hospital day (HD), the patient was transferred from the department of neurology to the department of rehabilitation. Conventional rehabilitation therapies, including physical therapy and occupational therapy, were provided twice per day, 5 times a week. On HD 44, the muscle tone of the paretic arm had increased to a modified Ashworth Scale (MAS) of 2 for the wrist, finger joints, and elbow joint, and antispastic medication (baclofen 30 mg/d) was prescribed. On HD 77, the muscle tone of the shoulder, elbow, hand, and fingers was aggravated to MAS 2 and 3, and the patient could not extend the clenched hand. One vial containing 200 U of neu-botulinium toxin A (neu-BoNT-A, Meditoxin, Medy-Tox, Ochang-eup, Korea) was diluted with 8 mL normal saline, and a total of 287.5 U was injected by 1 experienced physiatrist into the subscapularis, biceps brachii (Bi), brachioradialis (BR), brachialis (Bra), pronator teres, flexor carpi radialis (FCR), flexor digitorum profundus (FDP), flexor digitorum superficial (FDS), and flexor pollicis longus (FPL) muscles, under electrical stimulation and ultrasound guidance. There were no side-effects and 2 types of exoskeletal upper limb robot were then applied. The program of the Neuro-X system (Apsun Inc., Seoul, Korea) was progressed from passive mode to active-assisted exercises of the wrist, elbow, and shoulder joints in 2 dimensions through games that depended on the patient's functional status and that consisted of horizontal rotational movements at an angular velocity of 30 degrees per second, allowing shoulder abduction–adduction and elbow flexion–extension. The tasks of the ReoGo system (Motorika Medical Ltd, Israel) consisted of virtual reality-based execution of 3D movements with mobilization of the shoulder and elbow joints and hand gripping, with these movements resembling real ADL tasks (reaching and cup-to-mouth maneuvers). The movement mode can be progressed from completely passive to completely active, allowing the robot's arm to exert variable degrees of training (Fig. 1).

Significant improvements were observed in the manual muscle test (MMT), manual function test (MFT), box and block test (BBT), Korean version of the modified Barthel index (K-MBI), Fugl-Meyer Assessment (FMA-UL), and grip strength of the paretic arm during the rehabilitation therapy period (Table 1).

**Table 1:**
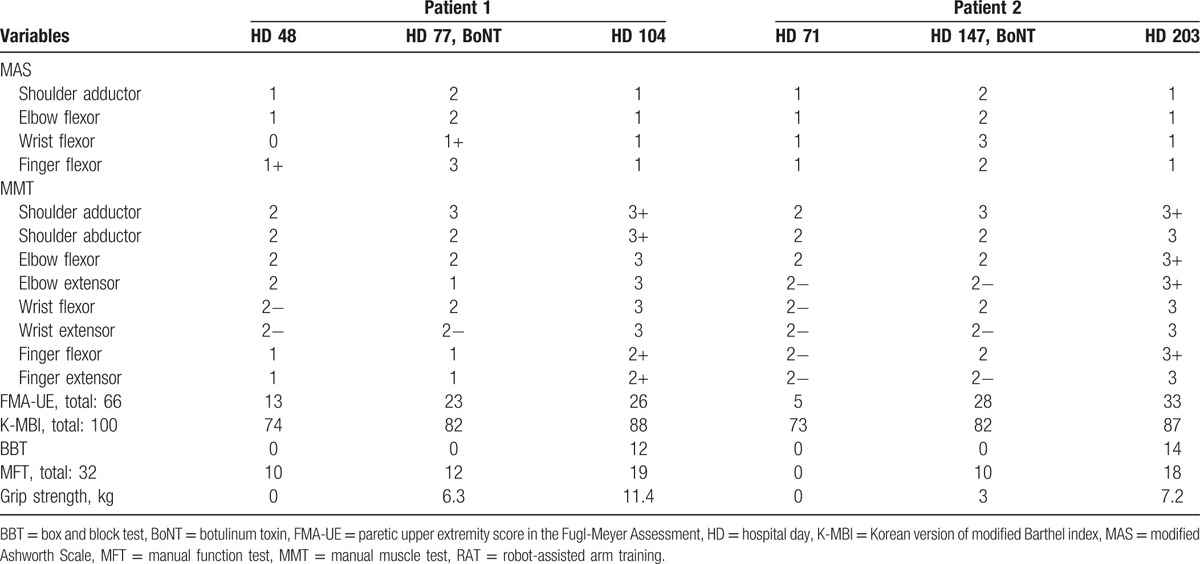
Alterations of clinical outcome measure after combined therapy of BoNT and RAT.

### 2.2 Patient 2

On October 28, 2015, a 51-year-old man visited the emergency room of OO hospital because of dysarthria, dysphagia, and right hemiparesis, and was diagnosed with intracranial hemorrhage in the left basal ganglia; stereotactic aspiration of an intracerebral hematoma was conducted. On HD 17, the patient was transferred from the department of neurology to the department of rehabilitation, and physical therapy, occupational therapy, and speech therapy were conducted. On HD 149, the muscle tone of the paretic shoulder, elbow, finger, and wrist had increased to MAS 2 and 3, but the patient could not extend the wrist. A total of 350 U neu-BoNT-A was injected into the subscapularis, Bi, BR, Bra, FCR, FDS, FDP, and FPL, and 2 types of exoskeletal robot-aided arm therapy were initiated (Fig. 1). Significant improvements were observed in the MMT, MFT, BBT, K-MBI, FMA-UL, and grip strength after multidisciplinary rehabilitation therapy (Table 1).

## 3 Discussion

In this case report, multidisciplinary rehabilitation therapies involving the use of RAT and a BoNT-A injection with conventional rehabilitation therapy during the subacute phase of stroke induced an improvement in hand strength and function in daily activities affected by upper limb spasticity. Although spasticity emerges and disappears according to motor recovery, chronic stroke patients usually sustain synergistic patterns of abnormal coactivation that flex the arm and hand if they try to extend them voluntarily; this is the result of spastic flexors combined with weak extensor muscles in the paretic arm and hand.^[5]^ Although the BoNT-A injection may weaken the strength of the spastic elbow, wrist, and finger flexors, it allows improvements to the release of the hand grip and reaching tasks of the elbow and wrist, and consequently leads to bimanual participation in daily activities.^[6]^ Furthermore, it may induce synaptic plastic reorganization at the muscular afferents, spinal motor neurons, interneuron system, and beyond,^[7]^ and facilitates neural plasticity and motor relearning through goal-oriented training programs.^[8]^

Precise and detailed injections to extensive arm muscles (including the shoulder adductor) in the subacute phase may have altered the natural process of motor recovery. However, the Copenhagen study reported that 80% of stroke patients had reached their best ADL function within 6 weeks postonset, and 95% had completed functional recovery within 12.5 weeks postonset. The Copenhagen 2 stroke durations of the patients in this report were 11 weeks and 21 weeks, and they were considered to have achieved the plateau of ADL functions at the BoNT-A injection. Indeed, high-intensity repetitive task-oriented robotic arm and hand training was performed to enhance neuroplasticity, and performances in BBT (manual dexterity) and handgrip strength were improved at discharge. Although the paretic arm functions were not fully satisfactory for the independent performance of ADL, they permitted increased chance of using the paretic upper limb in ADL, such as in grasping and holding objects or bimanual manipulation activities.

## 4 Conclusion

BoNT-A injection at the subacute stage and combined conventional rehabilitation therapy and RAT contributed to improvements in voluntary hand grasp power, manual dexterity, and spasticity. Therefore, a multidisciplinary rehabilitation treatment in combination with a BoNT-A injection and RAT should be recommended for enhancing appropriate neuroplasty in subacute spastic stroke patients.
